# Choroid Plexus Papilloma of the Fourth Ventricle in a Pediatric Patient: A Case Report

**DOI:** 10.7759/cureus.24892

**Published:** 2022-05-10

**Authors:** Luma Qutub, Bandar Y Sulaimani, Khalid T Alghamdi, Ahmed A Mulla, Hussam Kutub

**Affiliations:** 1 College of Medicine, Batargi Medical College, Jeddah, SAU; 2 Medicine, College of Medicine, King Saud Bin Abdulaziz University for Health Sciences, Jeddah, SAU; 3 Medicine, College of Medicine, King Saud Bin Abdulaziz University for Health Sciences, King Abdullah International Medical Research Centre, Jeddah, SAU; 4 Neurosurgery, Ministry of National Guards Health Affairs, Jeddah, SAU; 5 Consultant Neurosurgery, King Abdulaziz Medical City, Ministry of National Guards Health Affairs, Jeddah, SAU

**Keywords:** sub-occipital craniotomy, pediatric tumor, obstructive hydrocephalus, fourth ventricular tumor, choroid plexus papilloma

## Abstract

Choroid plexus papilloma (CPP) is a rare intraventricular tumor. The common locations of the tumor vary based on the age of the patient. It usually occurs in the supratentorial region in children, however in adult patients, these tumors commonly present in the infratentorial region. We are presenting a rare case of a pediatric patient with a two month history of decreased activity and loss of interest in his surroundings and gait imbalance. He underwent a suboccipital craniotomy and excision of a CPP in the fourth ventricle. In conclusion, CCP should be considered as part of the differential diagnosis of intracranial tumors when the clinical presentation and investigations are suggestive regardless of the location to avoid misdiagnosing it when it occurs in an uncommon location.

## Introduction

Choroid plexus papilloma (CCP) is one of the rare intraventricular tumors that accounts for 0.4 to 1% of brain tumors in adults and 1.5 to 6% in the pediatric population [1]. It is a tumor of neuroectodermal origin, which can be cured by a total tumor resection [2]. In adults, it commonly occurs in the infratentorial region, and the most common site is the fourth ventricle. However, in the pediatric population, it commonly occurs in the supratentorial region with the most common site being the lateral ventricle [3]. In addition to that, even though it can appear at any age, 70% of the cases in the pediatric population occur in children younger than two years old [1,4,5]. CCP is a benign disease in 80% of patients with a good prognosis [6]. In this study, we present a rare case of a child with choroid plexus papilloma in the fourth ventricle.

## Case presentation

**Figure 1:**
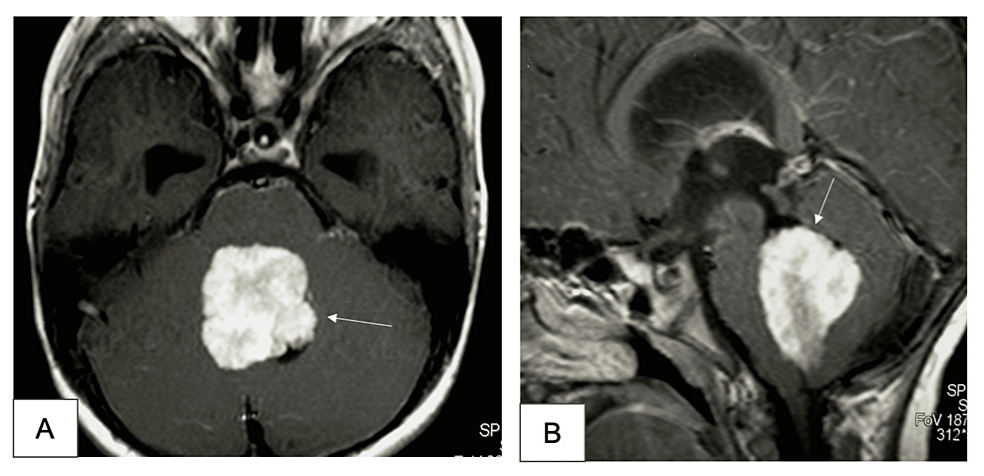
Axial (A) and sagittal (B) MRI images show a fourth ventricular tumor that lead to obstructive hydrocephalus MRI = magnetic resonance imaging

**Figure 2:**
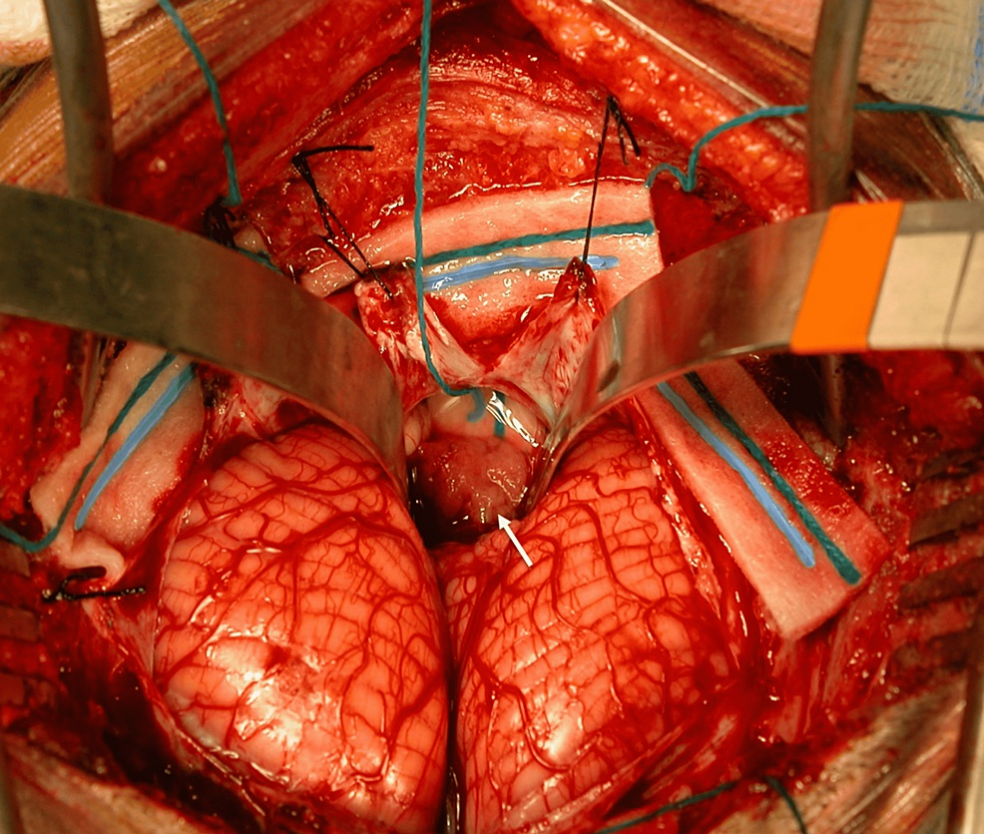
Sub-occipital craniotomy for excision of the fourth ventricular lesion

A seven-year-old boy presented with a two months history of decreased activity and loss of interest in his surroundings which was followed by a developing gait imbalance. On examination, the patient was alert and awake with no cranial nerve palsies, but he had dysdiadochokinesia and ataxia. A brain computed tomography (CT) and magnetic resonance imaging (MRI) showed a fourth ventricular enhancing lesion and hydrocephalus suggestive of a choroid plexus papilloma (Figure 1). The patient was taken to the operative room for a standard sub-occipital craniotomy with excision of the fourth ventricular lesion (Figure 2). The tumor was well demarcated arose from the choroid plexus of the fourth ventricle. A successful total excision was performed and no cerebrospinal fluid diversion. Post-operative imaging decomented total excision of the lesion and resolution of the hydrocephalus. Clinically, the patient demonstrated significant improvement in mood and communication. Upon discharge, the patient was conscious, alert, communicating and was able to sit independently and to walk with help.

## Discussion

The World Health Organization describes the classification of choroid plexus tumors as follows: low grade, atypical, and carcinoma, which is determined by histopathological examination. Choroid plexus papillomas have less than two mitotic figures per 10 high-power fields (HPFs), atypical ones have two to five per 10 HPFs, and carcinomas have more than five mitotic figures per 10 HPFs [7]. Benign choroid plexus tumors have a good prognosis after resection [2].

The clinical presentation of CPPs is mainly related to the raised intracranial pressure caused by the hydrocephalus which can cause headache with nausea or vomiting, reduced mentation and lateral gaze palsies. Subarachnoid hemorrhage may occur in some patients mainly due to bleeding from the tumor [8]. In our case, for instance, the patient presented with decreased activity, loss of interest in his surroundings, dysdiadochokinesia, and ataxia.

The differential diagnoses for choroid plexus papilloma include intraventricular tumors such as papillary ependymoma, medulloblastoma, and pilocytic astrocytoma among other intraventricular tumors [9]. Gross total resection is the treatment of choice. Hydrocephalus management is an important part of the treatment plan, which may require placing a ventriculoperitoneal (VP) shunt [10]. In our case, the patient did not require a VP shunt after the surgery. 

## Conclusions

Choroid plexus papilloma is a rare intracranial benign tumor that can cause hydrocephalus and increased intracranial pressure. In the pediatric population, the fourth ventricle is considered a rare location for CCPs. It is important to include CCP in the differential diagnosis of intracranial tumors in any location when the clinical presentation and imaging are supportive to avoid misdiagnosis if it occurred in a rare location. The treatment for CCP is complete surgical resection, which has an excellent prognosis and survival rate. Patients typically do not require any adjuvant treatment except in complicated or atypical cases.
